# Supplementary material for: Mapping nanoscale topographic features in thick tissues with speckle diffraction tomography
Source: Light Sci Appl. 2023 Aug 22;12:200. doi: 10.1038/s41377-023-01240-0 (PMC10444882; doi:10.1038/s41377-023-01240-0)
Supplement: Supplementary file 1 — Supplementary Information [file 41377_2023_1240_MOESM1_ESM.pdf]

Supplementary Information for
Mapping nanoscale topographic features in thick tissues with speckle diffraction tomography

Sungsam Kang[1], Renjie Zhou[2,*], Marten Brelen[3], Heather K. Mak[3], Yuechuan Lin[1], Peter T. C. So[1,4,5], and Zahid Yaqoob[1,6*]

[1]Laser Biomedical Research Center, G. R. Harrison Spectroscopy Laboratory, Massachusetts Institute of Technology, Cambridge, MA 02139, USA
[2] Department of Biomedical Engineering, The Chinese University of Hong Kong, Hong Kong, China
[3]Department of Ophthalmology and Visual Sciences, The Chinese University of Hong Kong, Hong Kong, China
[4]Department of Mechanical Engineering, Massachusetts Institute of Technology, Cambridge, MA 02139, USA
[5]Department of Biological Engineering, Massachusetts Institute of Technology, Cambridge, MA 02139, USA
[6]Department of Biomedical Engineering, Boston University, Boston, MA 02215, USA
*Correspondence: rjzhou@cuhk.edu.hk & zyaqoob@mit.edu

I. Working principle of speckle-correlation reflection phase microscopy (SpeCRPM)

As shown in Fig. S1, light from a supercontinuum laser (NKT Photonics, SuperK Extreme EXR-4) passes through a bandpass filter (centered at 800 nm with a bandwidth of 40 nm) followed by a rotating diffuser (RD) to generate a dynamic speckle-field. The dynamic speckle-field is then delivered to a Linnik-type interferometer that utilizes a polarization beam splitter (PBS) to split the incoming speckle field into sample and reference arms. A half-wave plate, denoted as λ/2 plate, is used to control the intensity ratio of sample and reference beams. For each arm of the interferometer, a quarter-wave plate, denoted as λ/4 plate, is inserted to guide the back-reflected field from both the reference mirror as well as from the sample into the detection path, where both fields are collinear but exhibit orthogonal linear polarization states. To realize off-axis holography, a diffraction grating (Ronchi Ruling, Edmund Optics) is placed at the intermediate image plane, from which +1 and -1 order diffracted beams are generated that represent the reference and the back-scattered sample fields, respectively, while their polarization states are maintained. Two polarizers with orthogonal transmission axes (P0 and P90) are used to select the reference field (+1 diffraction order) and back-scattered sample field (-1 diffraction order), respectively. At the final image plane, a camera (Flea3, Point Gray) is placed to record the interferograms. Since sample and reference beams have orthogonal polarization states, an additional polarizer (P45) with transmission axis at 45 degrees with respective to P0 and P90 is used to record the interference pattern. More detailed working principle and quantitative phase imaging demonstration about SpeCRPM can be found in Ref. [1].

With this setup, single-shot and wide-field quantitative phase imaging can be achieved with a maximum frame rate of 100 frames per second (fps), limited only by the camera speed and power spectral density of the light source. The high NA water immersion objective lens (60x/1.00 W, LUMPlanFL N, Olympus) and the 800 nm illumination source with $\Delta\lambda = 40$ nm spectral bandwidth (defined by a bandpass filter after the supercontinuum laser) enable us to obtain a lateral and axial spatial resolution of about 500 nm and 1 µm, respectively. For instance, as shown in the inset of Fig. S1, the raw interferogram shows a clear fringe contrast when the sample mirror is placed at $z = 0$. However, a dramatic reduction of contrast is observed with the sample mirror displaced by 1 µm along the axial direction, which verifies the high depth-sectioning ability of our system. Note that when utilizing the full spectrum of the supercontinuum laser, the temporal coherence response of the system shows multiple peaks [2] due to the spectral complexity of the supercontinuum laser, which makes it difficult to find the optimum time delay as discussed in our main text. After reducing the spectrum of the laser with a bandpass filter, the temporal coherence response of the system exhibits a single peak with a FWHM of 4 µm.

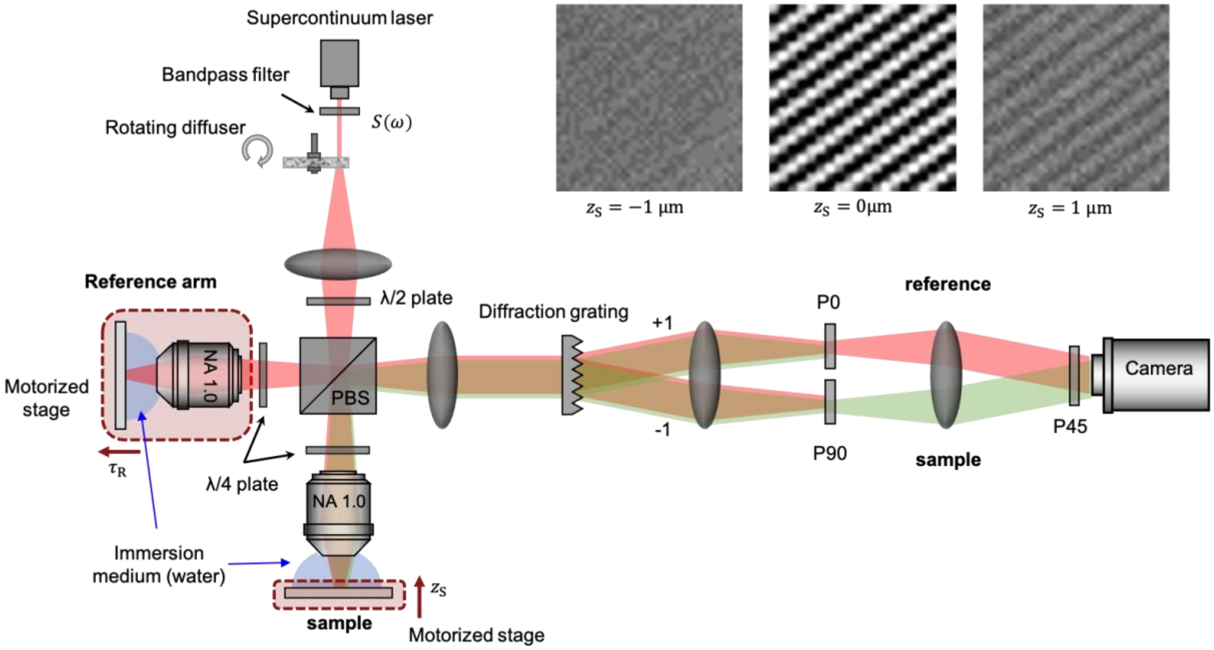

Fig S1. Schematic diagram of the SpeCRPM system. P0, P90, and P45 represent polarizers with transmission axes at 0, 90, 45 degrees, respectively. Beam in red color denotes the sample illumination field and reflected field from the reference mirror, while beam in green color denotes the back-scattered field from sample.

II. Theoretical framework SDT

Considering an arbitrary object, the scattered electric field U_s in space $\mathbf{r}$ can be described by the inhomogeneous wave equation as [3]:

$$\nabla^2 U_\mathrm{s}(\mathbf{r},\omega) + (n_0\omega/c)^2 U_\mathrm{s}(\mathbf{r},\omega) = -(\omega/c)^2 \chi(\mathbf{r}) U(\mathbf{r},\omega), \tag{S1}$$

where $\chi(\mathbf{r}) = n^2(\mathbf{r}) - n_0^2$ is the scattering potential of the object, $n(\mathbf{r})$ is the refractive index (RI) of the object, n_0 is the RI of the immersion medium, ω is the angular frequency of light, and c is the speed of light in free space. Here, we assume that χ does not change within the wavelength range of incident field. In general, the electric field U in the right-hand side of Eq. (S1) can be described as: $U = U_\mathrm{s} + U_\mathrm{i}$, where U_i is the incident field. Assuming the object is weakly-scattering, under the 1st order Born approximation, we have $U \simeq U_\mathrm{i}$ which implies that the incident field does not change inside the medium. This approach has been successfully applied to describing scattering inside cells and thin tissue sections [4]. However, when dealing with a thick object, such as a thick tissue section, this approximation is no longer valid as the incident field is modified inside the object due to a large phase-shift induced by the RI mismatch, which is discussed in detail in the following.

As illustrated in the right-hand side of Fig. 1a in the main text, due to refraction at each interface, the propagation angle of the incident field in the medium is changed. As a result of this effect, an optical pathlength difference or a phase shift is induced. This phase shift is angle dependent that we denote it as $\Delta\phi(\mathbf{k_r},\omega)$ in back aperture plane, where $\mathbf{k_r} = (k_x, k_y)$ is the in-plane momentum of the incident field with $k_r = |\mathbf{k_r}| = \sqrt{k_x^2 + k_y^2}$. Considering a single layer structure with an average RI of $\bar{n}$ and a thickness of d, this phase-shift can be calculated as:

$$\Delta\phi(\mathbf{k_r},\omega) = d\left(\sqrt{(\bar{n}\omega/c)^2 - k_r^2} - \sqrt{(n_0\omega/c)^2 - k_r^2}\right). \tag{S2}$$

This phase shift will modify the incident field in the medium, which can be expressed in the spatial frequency domain as [5]:

$$\widetilde{U}_{\mathrm{i}}'(\mathbf{k},\omega) = \gamma_{\mathrm{s}}\widetilde{U}_{\mathrm{i}}(\mathbf{k},\omega)\mathrm{e}^{i\Delta\phi(\mathbf{k_r},\omega)}, \tag{S3}$$

where $\widetilde{U}_{\mathrm{i}}$ is the Fourier transform of the incident field and $\widetilde{U}_{\mathrm{i}}'$ is the Fourier transform of the modified incident field inside the sample, and $\mathbf{k} = (k_x, k_y, k_z)$ is the wavevector in the 3D spatial frequency domain. Due to multiple scattering, the amplitude of the modified incident field decays with the scattering mean-free-path (SMF) l_{s} of the layer medium as $\gamma_{\mathrm{s}} = \exp[-d/2l_{\mathrm{s}}]$. Similar to the Born approximation, at depth d, we can approximate $\widetilde{U} = \widetilde{U}_{\mathrm{i}}' + \widetilde{U}_{\mathrm{s}} \simeq \widetilde{U}_{\mathrm{i}}' = \gamma_{\mathrm{s}}\widetilde{U}_{\mathrm{i}}(\mathbf{k},\omega)\mathrm{e}^{i\Delta\phi(\mathbf{k_r},\omega)}$ that takes into account that the incident light is modified by the angle-dependent phase shift inside the medium.

Next, let us consider one plane wave component $\mathbf{k}_i$ of the incident field, $\widetilde{U}_{\mathrm{i}}(\mathbf{k},\omega) = S(\omega)A(\mathbf{k_r})\delta(\mathbf{k}-\mathbf{k}_i)$, where $S(\omega)$ is the spectrum of incident light source, $A(\mathbf{k_r})$ is the complex amplitude of the plane wave component in spatial frequency domain, and $\mathbf{k}_i = \left(k_{xi},\ k_{yi}, k_{zi} = \sqrt{(n_0\omega/c)^2 - k_{xi}^2 - k_{yi}^2}\right)$ (note that $\mathbf{k}_{\mathbf{r},i} = (k_{xi}, k_{yi})$). Performing 3D spatial Fourier transforms over both sides in Eq. (S1), we have:

$$\begin{aligned}
[(n_0\omega/c)^2 - k^2]\widetilde{U}_{\mathrm{s}}(\mathbf{k},\omega) &= -(\omega/c)^2\tilde{\chi}(\mathbf{k})\otimes\widetilde{U}_{\mathrm{i}}'(\mathbf{k},\omega) \\
&= -(\omega/c)^2\int\tilde{\chi}(\mathbf{k}-\mathbf{k}')\,\gamma_{\mathrm{s}}S(\omega)A(\mathbf{k}_r')\delta(\mathbf{k}'-\mathbf{k}_i)\mathrm{e}^{i\Delta\phi(\mathbf{k}_r',\omega)}\mathrm{d}^3\mathbf{k}' \\
&= -\gamma_{\mathrm{s}}(\omega/c)^2 S(\omega)A(\mathbf{k}_{\mathbf{r},i})\tilde{\chi}(\mathbf{k}-\mathbf{k}_i)\mathrm{e}^{i\Delta\phi(\mathbf{k}_{\mathbf{r},i},\omega)},
\end{aligned} \tag{S4}$$

where $\otimes$ stands for the convolution operator. In experiment, we scan the sample axially with a translational stage. In other words, the object susceptibility χ is varying according to the position of the sample. If we define the axial position of the sample as z_S, we can write $\chi(\mathbf{r}') = \chi(x,y,z-z_S)$, with its Fourier transform $\mathcal{F}[\chi(\mathbf{r}')] = \tilde{\chi}(\mathbf{k})\mathrm{e}^{-ik_z z_S}$. Then, the single-scattered wave component can be solved as:

$$\begin{aligned}
\widetilde{U}_{\mathrm{s}}(\mathbf{k},\omega) &= \frac{\gamma_{\mathrm{s}}(\omega^2/c^2)S(\omega)A(\mathbf{k}_{\mathbf{r},i})\tilde{\chi}(\mathbf{k}-\mathbf{k}_i)\mathrm{e}^{-i(k_z-k_{zi})z_S}\mathrm{e}^{i\Delta\phi(\mathbf{k}_{\mathrm{r,i}},\omega)}}{k_z^2 - ((n_0\omega/c)^2 - k_r^2)} \\
&= -\gamma_{\mathrm{s}}(\omega^2/c^2)S(\omega)A(\mathbf{k}_{\mathbf{r},i})\tilde{\chi}(\mathbf{k}-\mathbf{k}_i)\mathrm{e}^{i\Delta\phi(\mathbf{k}_{\mathrm{r,i}},\omega)}\mathrm{e}^{-i(k_z-k_{zi})z_S} \\
&\quad\cdot\frac{1}{2q(\mathbf{k_r},\omega)}\left\{\frac{1}{k_z + q(k_r,\omega)} - \frac{1}{k_z - q(k_r,\omega)}\right\}
\end{aligned} \tag{S5}$$

with $k_r \equiv |\mathbf{k_r}| = \sqrt{k_x^2 + k_y^2}$, and $q(\mathbf{k_r},\omega) = \sqrt{(n_0\omega/c)^2 - k_r^2}$. After a 1D spatial inverse Fourier transform of $\widetilde{U}_{\mathrm{s}}(\mathbf{k},\omega)$, as described in Eq. (S5), with respect to k_z, we have:

$$\begin{aligned}
\widetilde{U}_{\mathrm{s}}(\mathbf{k_r},z,\omega) &= -\gamma_{\mathrm{s}}(\omega^2/c^2)S(\omega)A(\mathbf{k}_{\mathbf{r},i})\mathrm{e}^{i\Delta\phi(\mathbf{k}_{\mathrm{r,i}},\omega)} \\
&\quad\cdot\left\{\tilde{\chi}(\mathbf{k_r}-\mathbf{k}_{\mathrm{r,i}}, z - z_S)\mathrm{e}^{ik_{zi}(\omega)z}\right\}\otimes_z\left\{\frac{1}{2q(\mathbf{k_r},\omega)}\left[\mathrm{sgn}(z)\mathrm{e}^{-iq(\mathbf{k_r},\omega)z}\right.\right. \\
&\quad\left.\left. - \mathrm{sgn}(z)\mathrm{e}^{iq(\mathbf{k_r},\omega)z}\right]\right\},
\end{aligned} \tag{S6}$$

where $\otimes_z$stands for the convolution operator with respect to z. $\mathrm{sgn}(z)$ is the sign function whose value is +1 for positive z and -1 for negative z. Therefore, the back-scattered components U_{bs} at $z < 0$ can be obtained as:

$$\widetilde{U}_{\mathrm{bs}}(\mathbf{k_r}, z, \omega) = -\frac{\gamma_{\mathrm{s}}(\omega^2/c^2)S(\omega)A(\mathbf{k}_{\mathbf{r},\boldsymbol{i}})\mathrm{e}^{i\Delta\phi(\mathbf{k}_{\mathbf{r},\mathbf{i}},\omega)}}{2q(\mathbf{k_r},\omega)} \cdot \mathrm{e}^{-iq(\mathbf{k_r},\omega)z}\mathrm{e}^{i\{k_{zi}(\omega)+q(\mathbf{k_r},\omega)\}z_{\mathrm{S}}}\tilde{\chi}\left(\mathbf{k_r} - \mathbf{k}_{\mathbf{r},\mathbf{i}},\ -q(\mathbf{k_r},\omega) - k_{zi}(\omega)\right). \quad \text{(S7)}$$

As mentioned, Eq. (S7) describes the back-scattered field of modified incident field at depth of d. After backward propagation in the medium, the back-scattered field exhibits an additional phase shift $\Delta\phi(\mathbf{k_r},\omega)$. Note that the coordinate of $\Delta\phi(\mathbf{k_r},\omega)$ for both incident and backscattered fields are the same since we are dealing with lateral momentum $\mathbf{k_r}$.

At the camera plane, which is at a conjugated image plane of the sample plane, we can set $z = 0$. Also, there exists a maximum possible spatial frequency determined by the numerical aperture NA of objective lens as defined by the pupil function $P(\mathbf{k_r},\omega) = 1$ for $k_r < \omega NA/c$. Assuming unit magnification between the sample and camera planes, we can write the back-scattered field at the camera plane as:

$$\begin{aligned}\widetilde{U}^{\mathrm{D}}_{\mathrm{bs}}(\mathbf{k_r},\omega) &= \gamma_{\mathrm{s}}\widetilde{U}_{\mathrm{bs}}(\mathbf{k_r}, z = 0, \omega)P(\mathbf{k_r},\omega)\mathrm{e}^{i\Delta\phi(\mathbf{k_r},\omega)} \\ &= -\frac{\gamma_s^2\ (\omega^2/c^2)S(\omega)A(\mathbf{k}_{\mathbf{r},\boldsymbol{i}})\mathrm{e}^{i\Delta\phi(\mathbf{k}_{\mathbf{r},\mathbf{i}},\omega)}P(\mathbf{k_r},\omega)\mathrm{e}^{i\Delta\phi(\mathbf{k_r},\omega)}}{2q(\mathbf{k_r},\omega)} \\ &\quad \cdot \mathrm{e}^{i\{k_{zi}(\omega)+q(\mathbf{k_r},\omega)\}z_{\mathrm{S}}}\tilde{\chi}\left(\mathbf{k_r} - \mathbf{k}_{\mathbf{r},\mathbf{i}},\ -q(\mathbf{k_r},\omega) - k_{zi}(\omega)\right).\end{aligned} \quad \text{(S8)}$$

Next, we consider the interference due to a reference field U_{R} that is identical to the sample incident field U_{i} in the Linnik-type interferometer as:

$$\widetilde{U}^{\mathrm{D}}_{\mathrm{R}}(\mathbf{k_r},\omega) = S(\omega)A(\mathbf{k}_{\mathbf{r},\boldsymbol{i}})\delta(\mathbf{k_r} - \mathbf{k}_{\mathbf{r},\mathbf{i}})\mathrm{e}^{-i\omega\tau_{\mathrm{R}}} \quad \text{(S9)}$$

where $\delta(\,)$ is the Dirac delta function, and τ_{R} is the arrival time of the reference field. Let $z_{\mathrm{S}} = 0$ and $\tau_{\mathrm{R}} = 0$ indicate the top sample interface position and the arrival time from that interface, respectively. Note that the top sample surface is positioned at the focal plane of the objective lens. As illustrated in Fig. 1b of the main text, positive z_{S} corresponds to sample translation towards the objective lens, while positive τ_{R} corresponds to the relative time delay between sample and reference field. The total signal measured at the detector plane can be written as, $\left|U^{\mathrm{D}}_{\mathrm{bs}} + U^{\mathrm{D}}_{\mathrm{R}}\right|^2 = \left|U^{\mathrm{D}}_{\mathrm{bs}}\right|^2 + \left|U^{\mathrm{D}}_{\mathrm{R}}\right|^2 + U^{\mathrm{D}}_{\mathrm{bs}} \cdot U^{\mathrm{D}*}_{\mathrm{R}} + U^{\mathrm{D}*}_{\mathrm{bs}} \cdot U^{\mathrm{D}}_{\mathrm{R}}$. Then the interference term $U^{\mathrm{D}}_{\mathrm{bs}} \cdot U^{\mathrm{D}*}_{\mathrm{R}}$ in the transverse spatial frequency domain becomes:

$$\begin{aligned}\Gamma_{\mathrm{pl}}(\mathbf{k_r},\omega) &= \mathcal{F}\left[U^{\mathrm{D}}_{\mathrm{bs}} \cdot U^{\mathrm{D}*}_{\mathrm{R}}\right] \\ &= \widetilde{U}^{\mathrm{D}}_{\mathrm{bs}}(\mathbf{k_r},\omega) \otimes_{\mathbf{k_r}} \widetilde{U}^{\mathrm{D}*}_{\mathrm{R}}(-\mathbf{k_r},\omega) \\ &= \iint \widetilde{U}^{\mathrm{D}}_{\mathrm{bs}}(\mathbf{k}'_{\mathbf{r}},\omega)\widetilde{U}^{\mathrm{D}*}_{\mathrm{R}}(-\mathbf{k_r} + \mathbf{k}'_{\mathbf{r}},\omega)\mathrm{d}\mathbf{k}'_{\mathbf{r}} \\ &= S(\omega)^*A(\mathbf{k}_{\mathbf{r},\boldsymbol{i}})^*\mathrm{e}^{i\omega\tau_{\mathrm{R}}}\iint \widetilde{U}^{\mathrm{D}}_{\mathrm{bs}}(\mathbf{k}'_{\mathbf{r}},\omega)\,\delta(-\mathbf{k_r} + \mathbf{k}'_{\mathbf{r}} - \mathbf{k}_{\mathbf{r},\mathbf{i}})\mathrm{d}\mathbf{k}'_{\mathbf{r}} \\ &= S(\omega)^*A(\mathbf{k}_{\mathbf{r},\boldsymbol{i}})^*\mathrm{e}^{i\omega\tau_{\mathrm{R}}}\widetilde{U}^{\mathrm{D}}_{\mathrm{bs}}(\mathbf{k_r} + \mathbf{k}_{\mathbf{r},\mathbf{i}},\omega) \\ &= -\gamma_s^2\left(\frac{\omega^2}{c^2}\right)|S(\omega)|^2\left|A(\mathbf{k}_{\mathbf{r},\boldsymbol{i}})\right|^2\mathrm{e}^{i\Delta\phi(\mathbf{k}_{\mathbf{r},\mathbf{i}},\omega)}\mathrm{e}^{i\omega\tau_{\mathrm{R}}}\,\mathrm{e}^{ik_{zi}(\omega)z_S}\mathrm{e}^{iq(\mathbf{k_r}+\mathbf{k}_{\mathbf{r},\mathbf{i}},\omega)z_S} \\ &\quad \cdot \frac{P(\mathbf{k_r} + \mathbf{k}_{\mathbf{r},\mathbf{i}},\omega)\mathrm{e}^{i\Delta\phi(\mathbf{k_r}+\mathbf{k}_{\mathbf{r},\mathbf{i}},\omega)}}{2q(\mathbf{k_r} + \mathbf{k}_{\mathbf{r},\mathbf{i}},\omega)}\tilde{\chi}\left(\mathbf{k_r}, -q(\mathbf{k_r} + \mathbf{k}_{\mathbf{r},\mathbf{i}},\omega) - k_{zi}(\omega)\right).\end{aligned} \quad \text{(S10)}$$

The above equation assumes a monochromatic plane wave illumination with incident wavevector of $\mathbf{k}_{\mathbf{r},i}$, wavelength of $\lambda = 2\pi c/\omega$ and complex amplitude of $S(\omega)A(\mathbf{k}_{\mathbf{r},i})$. For our case of using a broadband dynamic speckle field for interference, we need to integrate Eq. (S10) over all possible incident wavevectors $\mathbf{k}_{\mathbf{r},\mathbf{i}}$ created by the rotating diffuser and all possible frequencies ω from the super-continuum laser after the bandpass filter. Assuming a uniform distribution for the speckle field in back-aperture plane, as limited by the pupil function P, we have $S(\omega)A(\mathbf{k}_{\mathbf{r},i}) = S(\omega)P(\mathbf{k}_{\mathbf{r},\mathbf{i}},\omega)$. Then, the final interference signal by the broadband dynamic speckle illumination becomes:

$$\begin{aligned}\Gamma(\mathbf{k}_\mathbf{r}) &= \iiint \Gamma_{\mathrm{pl}}(\mathbf{k}_\mathbf{r},\omega;\mathbf{k}_{\mathbf{r},\mathbf{i}})\, \mathrm{d}\mathbf{k}_{\mathbf{r},\mathbf{i}}\mathrm{d}\omega \\ &= -\left(\frac{\gamma_\mathrm{S}^2}{2c^2}\right)\iiint \omega^2|S(\omega)|^2\, e^{i\omega\tau_\mathrm{R}}\,\mathcal{A}(\mathbf{k}_\mathbf{r}+\mathbf{k}_{\mathbf{r},\mathbf{i}},z_\mathrm{S},\omega)\mathcal{B}^*(\mathbf{k}_{\mathbf{r},\mathbf{i}},z_\mathrm{S},\omega) \\ &\quad\cdot \tilde{\chi}\left(\mathbf{k}_\mathbf{r},\ -q(\mathbf{k}_\mathbf{r}+\mathbf{k}_{\mathbf{r},\mathbf{i}},\omega)-q(\mathbf{k}_{\mathbf{r},\mathbf{i}},\omega)\right)\mathrm{d}\mathbf{k}_{\mathbf{r},\mathbf{i}}\mathrm{d}\omega \end{aligned} \quad \text{(S11)}$$

with $\mathcal{A}(\mathbf{k}_\mathbf{r},z_\mathrm{S},\omega) = \frac{P'(\mathbf{k}_\mathbf{r},\omega)}{q(\mathbf{k}_\mathbf{r},\omega)}\mathrm{e}^{iq(\mathbf{k}_\mathbf{r},\omega)z_\mathrm{S}}$, $\mathcal{B}(\mathbf{k}_{\mathbf{r},\mathbf{i}},z_\mathrm{S},\omega) = P'^*(\mathbf{k}_{\mathbf{r},\mathbf{i}},\omega)\mathrm{e}^{-iq(\mathbf{k}_{\mathbf{r},\mathbf{i}},\omega)z_\mathrm{S}}$. Here we used $k_{zi} = q(\mathbf{k}_{\mathbf{r},\mathbf{i}},\omega)$ and defined a complex pupil function $P'(\mathbf{k}_{\mathbf{r},\mathbf{i}},\omega) = P(\mathbf{k}_{\mathbf{r},\mathbf{i}},\omega)\exp[i\Delta\phi(\mathbf{k}_\mathbf{r},\omega)]$ (note that $P(\mathbf{k}_{\mathbf{r},\mathbf{i}},\omega)^2 = P(\mathbf{k}_{\mathbf{r},\mathbf{i}},\omega)$ by the definition of the pupil function). According to Eq. (S11), the object is sampled on a curved surface in the 3D spatial spectrum domain as $\chi\left(\mathbf{k}_\mathbf{r},\ -q(\mathbf{k}_\mathbf{r}+\mathbf{k}_{\mathbf{r},\mathbf{i}},\omega)-q(\mathbf{k}_{\mathbf{r},\mathbf{i}},\omega)\right)$. Also, the integration over spectrum gives the inverse Fourier transform with respect to ω at $t = \tau_\mathrm{R}$. Assuming a point particle, i.e., letting $\tilde{\chi} = 1$, inside a layered medium with RI of $\bar{n}$ at depth d, the transfer function (TF) of the system can be finally obtained as a function of lateral spatial frequency $\mathbf{k}_\mathbf{r}$, sample position z_S, and temporal delay τ_R as:

$$\begin{aligned}\mathcal{T}(\mathbf{k}_\mathbf{r},z_\mathrm{S};\tau_\mathrm{R}) &= -\left(\frac{\gamma_\mathrm{s}^2}{2c^2}\right)\iiint \omega^2|S(\omega)|^2\, \mathrm{e}^{i\omega\tau_\mathrm{R}}\,\mathcal{A}(\mathbf{k}_\mathbf{r}+\mathbf{k}_{\mathbf{r},\mathbf{i}},z_\mathrm{S},\omega)\mathcal{B}^*(\mathbf{k}_{\mathbf{r},\mathbf{i}},z_\mathrm{S},\omega)\mathrm{d}\mathbf{k}_{\mathbf{r},\mathbf{i}}\mathrm{d}\omega \\ &= -\left(\frac{\gamma_\mathrm{S}^2}{2c^2}\right)\mathcal{F}_\omega^{-1}\left[\omega^2|S(\omega)|^2\left\{\mathcal{A}(\mathbf{k}_\mathbf{r},z_\mathrm{S},\omega)\star_{\mathbf{k}_\mathbf{r}}\mathcal{B}(\mathbf{k}_\mathbf{r},z_\mathrm{S},\omega)\right\}\right]\end{aligned} \quad \text{(S12)}$$

where $\star_{\mathbf{k}_\mathbf{r}}$ stands for the cross-correlation operator with respect to $\mathbf{k}_\mathbf{r}$. Note that Eq. (S12) is identical to Eq. (1) in the main text. By Fourier transform of Eq. (S12) with respect to z_S will lead to a 3D transfer function of SpeCRPM in 3D spatial frequency domain as described in Fig. 2 in the main text.

Next, the 3D point spread function (PSF) inside medium can obtained by taking an inverse Fourier transform of $\mathcal{T}(\mathbf{k}_\mathbf{r},z_\mathrm{S};\tau_\mathrm{R})$ as described in Eq. (S12) in $\mathbf{k}_\mathbf{r}$ space as:

$$\mathcal{P}(\mathbf{r},z_\mathrm{S};\tau_\mathrm{R}) = -\left(\frac{\gamma_\mathrm{S}^2}{2c^2}\right)\mathcal{F}_\omega^{-1}\left\{\omega^2|S(\omega)|^2\mathcal{F}_{\mathbf{k}_\mathbf{r}}^{-1}[\mathcal{A}(\mathbf{k}_\mathbf{r},z_\mathrm{S},\omega)]^*\cdot\mathcal{F}_{\mathbf{k}_\mathbf{r}}^{-1}[\mathcal{B}(\mathbf{k}_\mathbf{r},z_\mathrm{S},\omega)]\right\}. \quad \text{(S13)}$$

Using the circular symmetry, Eq. (S13) can be solved as a function of radial distance r as,

$$\mathcal{P}(r,z_\mathrm{S};\tau_\mathrm{R}) = -\left(\frac{\gamma_\mathrm{S}^2}{2c^2}\right)\mathcal{F}_\omega^{-1}\left\{\omega^2|S(\omega)|^2\mathcal{H}_{0,k_r}^{-1}[\mathcal{A}(k_r,z_\mathrm{S},\omega)]^*\cdot\mathcal{H}_{0,k_r}^{-1}[\mathcal{B}(k_r,z_\mathrm{S},\omega)]\right\} \quad \text{(S14)}$$

with $\mathcal{H}_{0,k_r}^{-1}[f(k_r)] = \frac{1}{2\pi}\int f(k_r)J_0(k_r r)k_r\mathrm{d}k_r$ is the inverse Hankel transform of the 0th order.

III. Focus shift and arrival time delay inside thick layered sample

The behavior of the focus shift and arrival time delay induced by thick layered sample can be investigated at the $\mathbf{k_r} = 0$ section of the transfer function $\mathcal{T}(\mathbf{k_r}, z_\mathrm{S}; \tau_\mathrm{R})$ as described in Eq. (S12) where the cross-correlation term becomes,

$$
\begin{aligned}
\mathcal{A}(\mathbf{k_r}, z_\mathrm{S}, \omega) \star_{\mathbf{k_r}} \mathcal{B}(\mathbf{k_r}, z_\mathrm{S}, \omega)\big|_{\mathbf{k_r}=\mathbf{0}} &= \iint \mathcal{A}(\mathbf{k}_\mathbf{r}', z_\mathrm{S}, \omega)\mathcal{B}^*(\mathbf{k}_\mathbf{r}', z_\mathrm{S}, \omega)\mathbf{dk}_\mathbf{r}' \\
&= \iint \frac{P(\mathbf{k}_\mathbf{r}', \omega)\mathrm{e}^{2i\Delta\phi(\mathbf{k}_\mathbf{r}',\omega)}}{q(\mathbf{k}_\mathbf{r}', \omega)} \mathrm{e}^{2iq(\mathbf{k}_\mathbf{r}',\omega)z_\mathrm{S}}\mathbf{dk}_\mathbf{r}' \\
&= 2\pi \int \frac{P(k_r', \omega)\mathrm{e}^{2i\Delta\phi(k_r',\omega)}}{q(k_r', \omega)} \mathrm{e}^{2iq(k_r',\omega)z_\mathrm{S}} k_r' \mathrm{d}k_r' \\
&= 2\pi \int \frac{P(k_r', \omega)\mathrm{e}^{2i\Delta\phi(k_r',\omega)}}{q(k_r', \omega)} \mathrm{e}^{2iq(k_r',\omega)z_\mathrm{S}} k_r' \cdot \frac{q(k_r', \omega)}{-2k_r'} \mathrm{d}(2q) \\
&= -\pi \int P(k_r', \omega)\mathrm{e}^{2i\Delta\phi(k_r',\omega)}\mathrm{e}^{iKz_\mathrm{S}}\mathrm{d}K \\
&= -\pi\mathcal{F}_K^{-1}\left\{P_K(K, \omega)\mathrm{e}^{2i\Delta\phi(k_r',\omega)}\right\}. \qquad \text{(S15)}
\end{aligned}
$$

In the above formulation, we used the circular symmetry and change of variable with $K = 2q(k_r', \omega)$. $\mathcal{F}_K^{-1}\{\cdot\}$ is inverse Fourier transform with respect to K. Also, we used the fact that the aperture function $P(\mathbf{k}_\mathbf{r}', \omega)$ and $\phi(\mathbf{k}_\mathbf{r}', \omega)$ are functions of $(\mathbf{k}_\mathbf{r}', \omega)$ independent to the polar angle in the polar coordinate. With the change of variable, we can define an axial aperture function $P_K(K, \omega)$ and axial phase shift function $\Delta\phi_K(K, \omega)$ as follows:

$$P_K(K, \omega) = 1 \ \text{ for} \sqrt{n_0^2 - NA^2} \le cK/2\omega \le n_0 \qquad \text{(S16)}$$

$$\Delta\phi_K(K, \omega) = d\left\{\sqrt{(K/2)^2 + (\bar{n}^2 - n_0^2)\,\omega^2/c^2} - K/2\right\} \qquad \text{(S17)}$$

Finally, the $\mathbf{k_r} = 0$ section of TF can be written as,

$$\mathcal{T}(\mathbf{k_r} = \mathbf{0}, z_\mathrm{S}; \tau_\mathrm{R}) = \left(\frac{\pi}{2c^2}\right)\mathcal{F}_\omega^{-1}\mathcal{F}_K^{-1}\left\{\omega^2|S(\omega)|^2 P_K(K, \omega)\exp\left[2i\Delta\phi_K(K, \omega)\right]\right\}, \qquad \text{(S18)}$$

which is an inverse Fourier transform of $\omega^2|S(\omega)|^2 P_K(K, \omega)\exp[2i\Delta\phi_K(K, \omega)]$ with respect to ω and K. From the translation property of Fourier transform, linear phase slope of the last term in the Eq. (S18) results in a displacement of $\left.\frac{\partial 2\Delta\phi_K}{\partial K}\right|_{K_p,\omega_p}$ in the spatial domain or as a focus shift Δf in the experiment. ω is transformed into time into as the reference arrival time τ_R in experiment.

As discussed in the main text, the focus shift and temporal shift are associated with propagation angle inside medium. Considering a plane wave component with propagation angle θ_p and angular frequency ω_p and letting $K_p = 2\omega_p n_0 \cos\theta_p / \mathrm{c}$, the resulting focus shift Δf can be obtained as:

$$\Delta f = \left.\frac{\partial 2\Delta\phi_K}{\partial K}\right|_{K_p,\omega_p} = d\left\{\frac{K}{\sqrt{K^2 + 4(\bar{n}^2 - n_0^2)\,\omega^2/c^2}} - 1\right\}_{K_p,\omega_p} = \frac{n_0 d\cos\theta_p}{\sqrt{\bar{n}^2 - n_0^2\sin^2\theta_p}} - d. \qquad \text{(S19)}$$

The temporal shift $\Delta\tau$ is obtained as:

$$\Delta\tau = \frac{\partial 2\Delta\phi}{\partial\omega}\bigg|_{K_p,\omega_p} = \left\{\frac{4d(\bar{n}^2 - n_0^2)\omega/c^2}{\sqrt{K^2 + 4(\bar{n}^2 - n_0^2)\,\omega^2/c^2}}\right\}_{K_p,\omega_p} = \frac{2d(\bar{n}^2 - n_0^2)/c}{\sqrt{\bar{n}^2 - n_0^2\sin^2\theta_p}}. \quad \text{(S20)}$$

From Eqs. (S19) and (S20), we find that the focus shift and temporal shift are functions of propagation angle θ_p. In experiments, illumination and detection process are related to multiple angles. Thus, the actual focus and temporal shifts should be determined by averaging all the plane-wave components within numerical aperture α.

Considering the maximum propagation angle limited by the numerical aperture of the objective lens, i.e., $n_0 \sin\theta_p = NA$, the location of shifted focus z_S can be obtained as:

$$z_S = d + \Delta f = d\sqrt{\frac{n_0^2 - NA^2}{\bar{n}^2 - NA^2}}, \quad \text{(S21)}$$

which coincides with conventional focus tracking method [6]. In addition, for a specific propagation angle θ_p, the ratio between pathlength shift $c\Delta\tau$ and shifted focus position z_S can be written as:

$$\eta(n) = \frac{c\Delta\tau}{z_S} = \frac{2(\bar{n}^2 - n_0^2)}{n_0\cos\theta_p}, \quad \text{(S22)}$$

As discussed in the main text, this ratio is a function of propagation angle and average RI of the medium, but independent to the layer thickness.

In the above discussion, we assumed a single propagation angle inside the sample to estimate the focus shift Δf and arrival time delay $\Delta\tau$. The actual Δf and $\Delta\tau$ are obtained by integrating all the possible propagation angles within the numerical aperture. Note that there are no closed-forms for Δf and $c\Delta\tau$, but they can be numerically obtained by tracking the peak positions of the PSF, as described in Eq. (S14), which is further illustrated in Fig. S2a-b. In experiments, once the shifted location of PSF is measured, the RI (n) and thickness (d) can be inversely recovered with the one-to-one correspondence between (n, d) and $(\Delta\tau, z_S)$. For instance, with the numerical results as plotted in Fig. S2a-b, we can generate a look-up table to retrieve n and d, as illustrated in Fig. 5 (see the Method section of the main text).

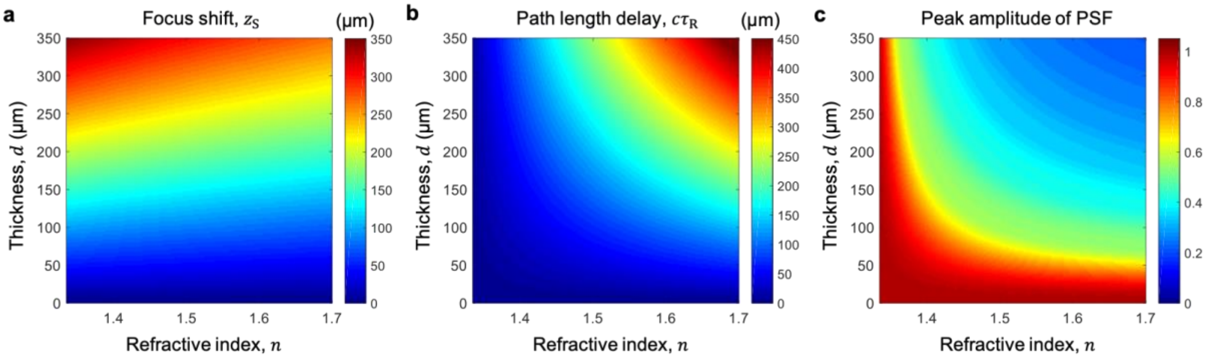

Fig S2. Behavior of PSF formed inside a thick sample. a-b Map of shifted focus location (z_S, $c\Delta\tau$) numerically tracked as a function of medium thickness and RI with assuming numerical aperture $\alpha = 1$, center wavelength $\lambda = 800$ nm, and spectral bandwidth $\Delta\lambda = 40$ nm. **c** Map of peak amplitude of the PSF in the medium normalized by the peak amplitude of the ideal PSF.

In addition, we can investigate the behavior of amplitude attenuation of the back-scattered field by the spherical aberration by simulating the PSF. As presented in Fig. 2 of the main text, the PSF inside medium is elongated and distorted. As a result, the peak amplitude of PSF decreases as the average RI and thickness of the medium increase, as illustrated in Fig. S2c. When dealing with a scattering sample, such as thick tissues, the scattering mean free path (SMF) is used to describe the sample scattering property. SMF can be used to quantify the attenuation of light intensity by multiple scattering inside the sample. However, the signal attenuation in a thick scattering medium is determined by both scattering and spherical aberration. With the simulation result in Fig. S2c, we can identify the contribution of aberration to the signal decay, thus obtaining a more precise scattering parameter.

IV. Scan strategy for optimizing reference path length delay

To retrieve RI and thickness in experiments, we need to find the optimum reference pathlength $\tau_{\mathrm{R}} = \Delta\tau$ which maximizes the interference signal at a depth z_{S}. However, scanning the reference pathlength will also result in collecting signal from other depths, thus making it difficult to find the optimum $\Delta\tau$ when dealing with scattering samples. According to our SDT model, there is a relation between the optical thickness nd and the location of PSF. As shown in Fig. S3, the contour lines of optical thickness nd have a linear relation between z_{S} and $\Delta\tau$. In other words, if we scan both the sample position z_{S} and reference pathlength τ_{R} following the contour line of a certain optical thickness during the experiment, we can selectively collect signal from the optical depth of the sample. Then, we can retrieve RI and thickness from the signal maxima on the contour line with the look-up table as illustrated in Fig. 5.

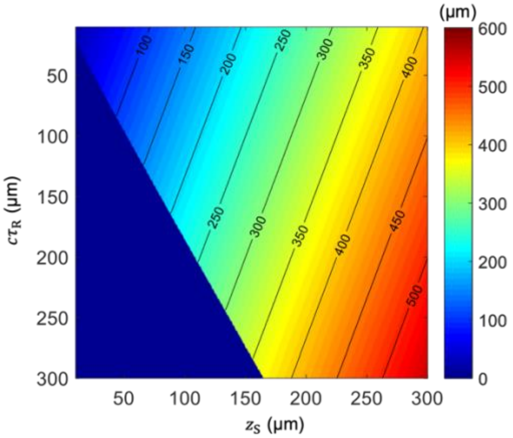

Fig S3. Illustration of optical thickness nd as a function of ($z_{\mathrm{S}}, \tau_{\mathrm{R}}$). Solid lines correspond to the contour lines of constant optical thickness.

V. Refractive index characterization using a PDMS structure

To verify the SDT model, we first studied a wedge-shaped structure (with apex angle ~3.4º) made from Polydimethylsiloxane (PDMS with uniform RI, n~1.41) material as shown in Fig. S4a. We measured the RI and the thickness of the PDMS sample at 3 different locations, marked as (1), (2), and (3) with SpeCRPM system. Since the sample has two distinct interfaces, i.e., the top water-PDMS interface and the bottom PDMS-glass interface, we measured two one-dimensional (1D) PSFs corresponding to the top and bottom interfaces by measuring the back-scattered complex fields at each interface while scanning z_{S}. Figure S4b-c show the two experimental PSFs (shown in red) for location (1), measured at $z_{\mathrm{S}} = (0, 290.4)$ μm with corresponding $c\tau_{\mathrm{R}} = (0, 104.8)$ μm. Next, we performed the same measurements for locations (2) and (3).

Figure S4d shows the corresponding PSF locations in the (z_S, τ_R) space for the bottom interface (3 red dots) at positions (1), (2) and (3) with respect to the top interface (blue dot). All three PSF positions, corresponding to the bottom interface, are mapped on the dashed line that has a slope $\eta(n) = 0.36$. Next, we retrieve n and d using the look-up table $L(z_S, \tau_R)$. As illustrated in Figs. S4e-f, while the thickness varies as a function of location as expected due to the wedge-shaped geometry of the sample, the RI remains unchanged and is measured to be ~1.41, which is in a good agreement with the optical property of PDMS. Furthermore, the retrieved RI and thickness value for location (1), when substituted in Eq. (3), enables obtaining theoretical PSFs (blue curves in Figs. S4b-c), which also match well with the corresponding experimental PSFs.

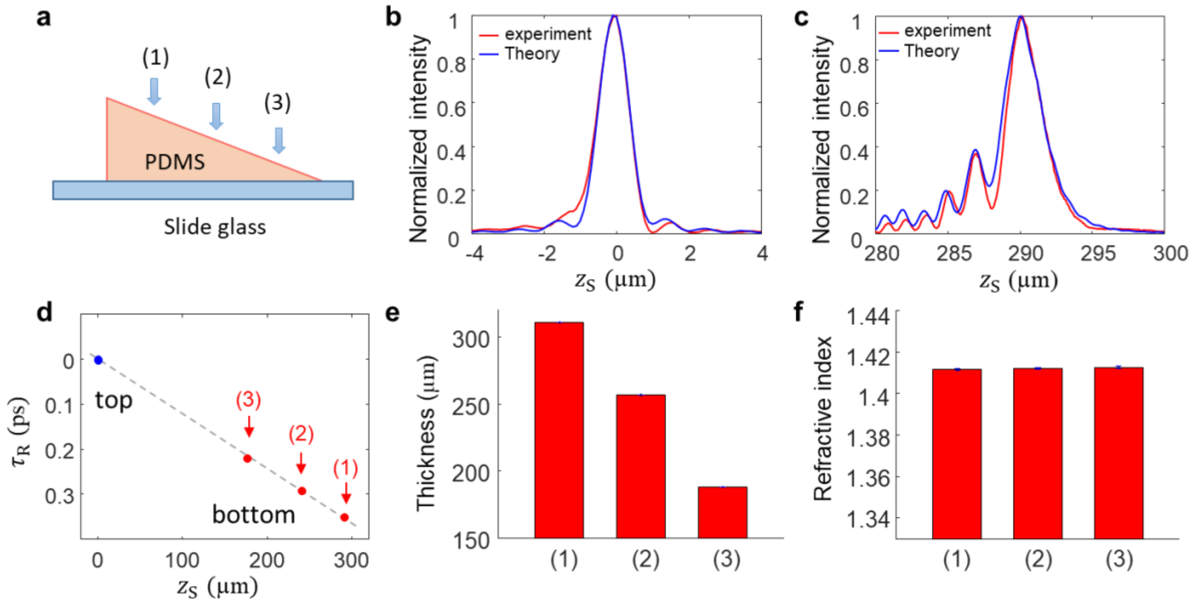

Fig S4. Refractive index measurements on a wedge-shaped structured made from PDMS. a Illustration of the wedge-shaped PDMS structure. The measurements were performed at 3 different positions, labeled as (1), (2) and (3). **b-c** Measured 1D PSF along the axial direction for top and bottom interfaces at position (1). Red curves show the experimentally measured PSFs, whereas the blue curves represent the corresponding theoretical PSFs. **d** PSF locations in the (z_S, τ_R) space for the bottom interface (shown as red dots) for positions (1), (2) and (3) with respect to the top interface (shown as blue dot). **e-f** Thickness (**e**) and RI (**f**) for the 3 locations in (**a**).

VI. Validation of imaging resolution and phase measurement accuracy

In this section, we validate the performance of SpeCRPM and SDT framework by using standard resolution targets. We first used a commercially available phase resolution target (Benchmark Tech.), as depicted in Fig. S5. From the phase map in Fig. S5b, we can identify line pairs up to the 4th element of group 10 in the resolution target, which has a designed line width of 345 nm and a separation of 690 nm between lines. Furthermore, from the line profile depicted in Fig. S5c, the height of the target patterns is estimated to be ~ 106 nm, which agrees well with both the designed height of ~ 100 nm and the AFM measurement of ~ 104.2 nm as provided by the manufacturer.

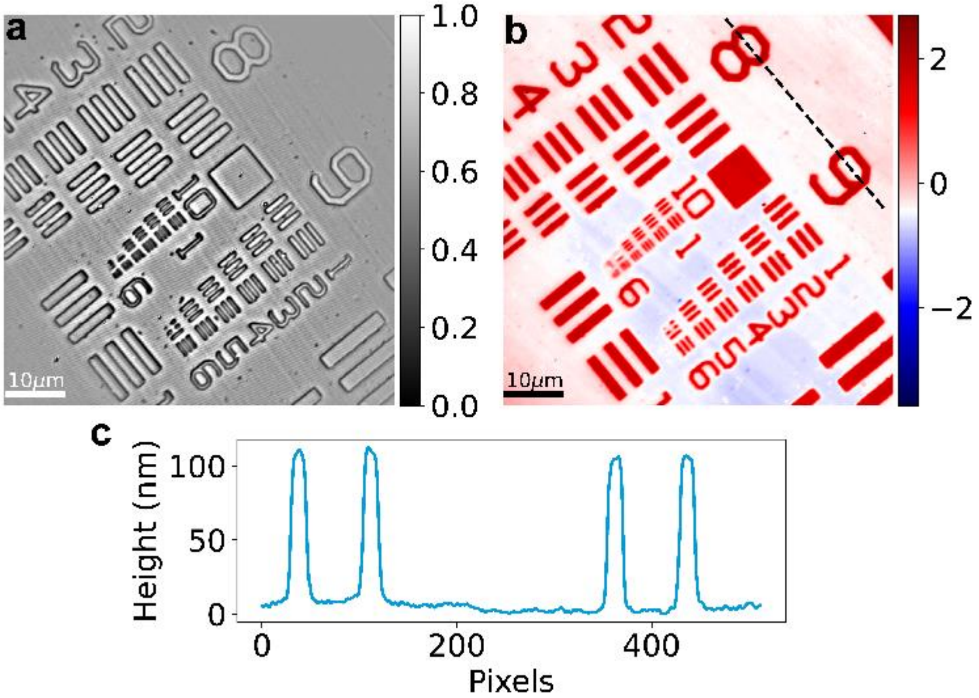

Fig S5. Validation of SDT system with a phase resolution target. a-b Normalized intensity and phase map of commercial phase resolution target measured by SDT system. **c** Height profile across the dashed line in (**b**) after subtracting the minimum value.

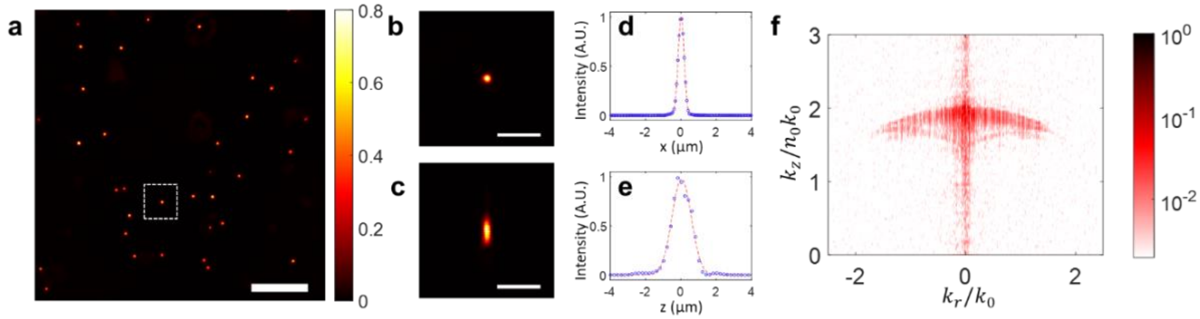

Fig S6. Validation of SDT system with gold nanoparticles. a Intensity map of gold nanoparticles (150 nm) distributed across ROI. Scale bar, 10 µm. **b** Magnified intensity map of gold nanoparticle inside the dashed box in **a**. Scale bar, 3 µm. **c**, x-z section image of gold nanoparticle in (**b**). Scale bar, 3 µm. **d** Cross-section of **b** (blue dots) along x-axis, and Gaussian fit (red dashed line). **e** Cross-section of **c** (blue dots) along z-axis, and Gaussian fit (red dashed line). **f** Cross-section of object spectrum in $(k_r - k_z)$ space. Color scale is adjusted to log scale for better visualization.

Next, we imaged 150 nm gold nanoparticles to confirm both the lateral and axial resolution of the system. A complex image stack of the particles was obtained by performing an axial scan. Figure S6a shows the intensity map of the central section of the complex image stack. For a specific particle located inside the dashed box of Fig. S6a, we show the horizontal and vertical cross-section images in Fig. S6b and Fig. S6c, respectively. The corresponding line profiles are presented in Fig. S6d and Fig. S6e, respectively. From the

line profiles, we determined a full width half maximum (FWHM) of 411 nm and 1.23 μm along the x and z axes, respectively. Considering the size of the gold particle, the numbers are in good agreement with the lateral and axial FWHM values of the intensity PSF as calculated in Fig. 2b and Fig. 2e in the main text. Moreover, by applying a 3D Fourier transform over the complex image stack, we recovered the spatial frequency spectrum of the gold nanoparticles, which closely matches the theoretical estimation shown in Fig. 2c.

VII. Refractive index measurements on a rat corneal tissue

Volumetric images of the Sprague Dawley (SD) rat corneal tissue sample were acquired by obtaining depth-resolved wide-field images at different depth using SpeCRPM. Figure S7a shows a cross-sectional image of the measured volume acquired by scanning the sample over 250 μm with a step size of 400 nm. The scanned depth range was manually divided into 6 regions as guided by the features in Fig. S7a. We experimentally optimized the interference signal by tuning τ_R at each interface separating different regions, thus providing corresponding locations of the PSF in the (z_S, τ_R) space. The slope of individual line segments connecting different dots (representing interfaces) in Fig. S7d is used to determine the average RI and thickness of the corresponding regions using the look-up table, as shown in Fig. S7e. The RI mapping of the cornea tissue clearly identifies 3 distinct layers, namely epithelium, stroma, and endothelium with $n = (1.37, 1.35, \text{and } 1.39)$ and corresponding thickness $d = (32, 138, \text{and } 8)$ μm, respectively.

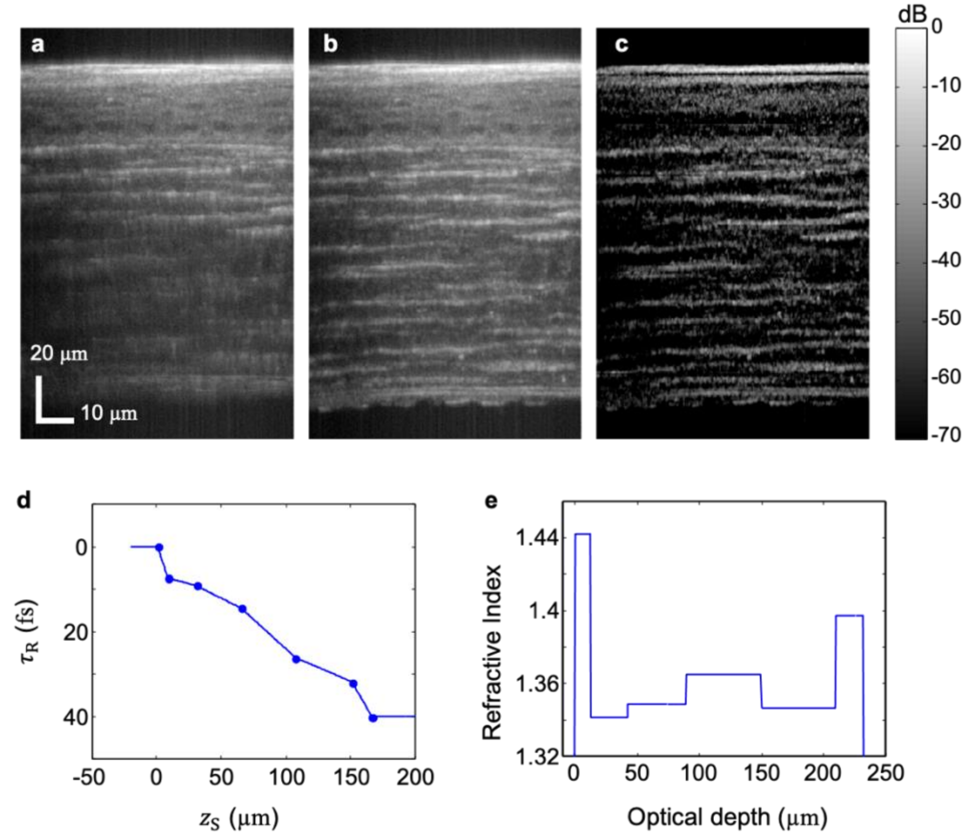

Fig S7. Volumetric image and refractive index measurement of a rat corneal tissue. **a** Normalized cross-sectional intensity image of the corneal tissue from a Sprague Dawley (SD) rat without reference pathlength correction. **b** Normalized cross-sectional intensity image with reference pathlength correction, according to (z_S, τ_R) relation shown in (**d**). **c** 3D deconvolution of (**b**) with the 3D PSF, calibrated by the RI

in **e**, based on the Richardson-Lucy algorithm. We used log-scale colormaps for (**a**-**c**) as $20 \times \log{(I/I_{\max})}$ (in dB units) for better visualization of deeper tissue region. **d-e** Signal distribution in the $(z_{\mathrm{S}}, \tau_{\mathrm{R}})$ space and the corresponding depth-resolved RI.

Subsequently, we obtained improved volumetric image of the corneal tissue sample by correcting the temporal shift induced by the sample itself. Figure S7b shows the corresponding cross-sectional image by scanning both the sample as well as the reference pathlength, according to Fig. S7d. At shallow depths, both Fig. S7a and S7b show similar structures and contrast since the temporal delay is not significant. However, as we go deeper into the cornea tissue, the temporal delay becomes significant. Therefore, a dramatic improvement in image contrast is achieved after the reference pathlength correction.

In addition, we computed the PSF along the depth of the cornea tissue according to the measured RI distribution. Next, we performed a 3D deconvolution based on Richardson-Lucy algorithm to further improve the volumetric image contrast and resolution. Specifically, we divided the whole volume into multiple 20 μm-thick sub-volumes along the depth and perform 3D deconvolution on each sub-volume with the corresponding PSF. Finally, all the deconvolved sub-volumes are stitched together to render the whole volume as shown in Fig. S7c, which shows improved image contrast as well as higher axial resolution.

VIII. Comparison with other imaging modalities

The working principle of the SDT system shares some similarities with OCT and full-field OCT (FF-OCT). However, our system offers several key advantages:

1. Compared to FF-OCT, which typically requires 2 to 4 phase-stepping images for acquisition, our SDT system utilizes an off-axis configuration that allows for single-shot measurements. This not only significantly improves data acquisition speed but also mitigates the degradation of interference contrast due to the phase fluctuation of the sample specimen during the phase stepping. Consequently, our system offers advantages over FF-OCT in terms of speed and image contrast.
2. In FF-OCT, the multiple scattering of sample can lead to significant speckle noise and crosstalk, resulting from the interference between adjacent pixels at the camera plane [7]. To avoid these challenges, FF-OCT typically utilize spatially incoherent light sources such as thermal sources. However, the limited brightness per spatial mode of such incoherent light introduces a trade-off between sensitivity and acquisition speed. On the contrary, dynamic speckle illumination with randomized phase distribution in the SDT system enables decorrelation of multiple-scattered light that inherently mitigates the speckle noise and crosstalk without compromising the sensitivity and acquisition speed.
3. In OCT and FF-OCT, the estimation of 1D RI is achievable through the focus tracking method, considering the maximum incidence angle within the framework of ray optics geometry. In contrast, SDT addresses the inverse scattering problem by solving the wave equation, allowing for the consideration of all incidence angles of illumination to offer a more accurate quantification of RI. Moreover, the inverse scattering model employed in SDT can be potentially applied to extend the 1D RI estimation to full 3D RI determination, as discussed in the Summary and outlook section as a direction for our future work.

These advantages distinguish the SDT system from FF-OCT and contribute to its enhanced performance and capabilities. When compared to other reflection-mode QPI methods, SDT offers several distinct advantages, such as fast imaging speed enabled by the single-shot measurement technique and high 3D

spatial resolution (lateral ~500 nm and axial ~1 μm) achieved by speckle correlation. We have conducted a comparison with those presented in selected representative papers, as summarized in Table 1.

Table 1. Specification of SDT compared to reported reflection mode QPI methods

	**SDT**	**en face OCT Ref. [39]**	**Epi-GLIM Ref. [40]**	**AO-SASM Ref. [41]**
**Frame rate (Hz)**	100	10	4	4
**Lateral resolution (μm)**	~ 0.5	> 3	~ 0.5	~ 0.4
**Axial resolution (μm)**	~ 1.2	~ 4.5	~ 1.5	~ 2
**Additional Features**	Full-field; single-shot measurement; quantifying depth-resolved RI.	In-vivo retinal imaging; computational adaptive optics	Compact module integrable with commercial microscopes	Computational adaptive optics; *in vivo* 3D imaging

References

1. Choi, Y., et al., *Dynamic speckle illumination wide-field reflection phase microscopy*. Optics Letters, **39**(20) 6062-6065 (2014).
2. Choi, Y., et al., *Reflection phase microscopy using spatio-temporal coherence of light.* Optica, **5**(11) 1468-1473 (2018).
3. Wolf, E., *Three-dimensional structure determination of semi-transparent objects from holographic data.* Optics Communications, **1**(4) 153-156 (1969).
4. Jin, D., et al., *Tomographic phase microscopy: principles and applications in bioimaging [Invited].* J Opt Soc Am B, **34**(5) B64-B77 (2017).
5. Booth, M.J., M.A. Neil, and T. Wilson, *Aberration correction for confocal imaging in refractive-index-mismatched media.* Journal of microscopy, **192**(2) 90-98 (1998).
6. Tearney, G., et al., *Determination of the refractive index of highly scattering human tissue by optical coherence tomography.* Optics letters, **20**(21) 2258-2260 (1995).
7. Subhash, H.M., *Full-field and single-shot full-field optical coherence tomography: a novel technique for biomedical imaging applications.* Advances in Optical Technologies, Vol. 2012, 435408 1-26 (2012).
